# Anti-Inflammatory Activity of Fucan from *Spatoglossum schröederi* in a Murine Model of Generalized Inflammation Induced by Zymosan

**DOI:** 10.3390/md21110557

**Published:** 2023-10-26

**Authors:** Ana Katarina Andrade Silva, Cássio Ricardo de Medeiros Souza, Hylarina Montenegro Diniz Silva, Jéssica Teixeira Jales, Lucas Alves de Souza Gomez, Ericka Janine Dantas da Silveira, Hugo Alexandre Oliveira Rocha, Janeusa Trindade Souto

**Affiliations:** 1 Department of Microbiology and Parasitology, Department of Biochemistry, Federal University of Rio Grande do Norte, Avenida Salgado Filho, BR 101, Campus Universitario, Lagoa Nova, Natal 59078-900, Brazil; katarina_andrade@hotmail.com (A.K.A.S.); cassiormsouza@gmail.com (C.R.d.M.S.); hylarina.silva@ebserh.gov.br (H.M.D.S.); jessica_jales@hotmail.com (J.T.J.); lucas.gomez.047@ufrn.edu.br (L.A.d.S.G.); hugo.rocha@ufrn.br (H.A.O.R.); 2 Onofre Lopes University Hospital, Federal University of Rio Grande do Norte, EBSERH, Natal 59078-900, Brazil; 3 Biochemistry and Molecular Biology Post-Graduation Program, Federal University of Rio Grande do Norte, Avenida Salgado Filho, BR 101, Campus Universitario, Lagoa Nova, Natal 59078-900, Brazil; 4 Department of Dentistry, Federal University of Rio Grande do Norte, Avenida Salgado Filho, 1787, Lagoa Nova, Natal 59056-000, Brazil; ericka_janine@yahoo.com.br

**Keywords:** inflammation, fucan, *Spatoglossum schröederi*, zymosan

## Abstract

Fucans from marine algae have been the object of many studies that demonstrated a broad spectrum of biological activities, including anti-inflammatory effects. The aim of this study was to verify the protective effects of a fucan extracted from the brown algae *Spatoglossum schröederi* in animals submitted to a generalized inflammation model induced by zymosan (ZIGI). BALB/c mice were first submitted to zymosan-induced peritonitis to evaluate the treatment dose capable of inhibiting the induced cellular migration in a simple model of inflammation. Mice were treated by the intravenous route with three doses (20, 10, and 5 mg/kg) of our fucan and, 1 h later, were inoculated with an intraperitoneal dose of zymosan (40 mg/kg). Peritoneal exudate was collected 24 h later for the evaluation of leukocyte migration. Doses of the fucan of *Spatoglossum schröederi* at 20 and 10 mg/kg reduced peritoneal cellular migration and were selected to perform ZIGI experiments. In the ZIGI model, treatment was administered 1 h before and 6 h after the zymosan inoculation (500 mg/kg). Treatments and challenges were administered via intravenous and intraperitoneal routes, respectively. Systemic toxicity was assessed 6 h after inoculation, based on three clinical signs (bristly hair, prostration, and diarrhea). The peritoneal exudate was collected to assess cellular migration and IL-6 levels, while blood samples were collected to determine IL-6, ALT, and AST levels. Liver tissue was collected for histopathological analysis. In another experimental series, weight loss was evaluated for 15 days after zymosan inoculation and fucan treatment. The fucan treatment did not present any effect on ZIGI systemic toxicity; however, a fucan dose of 20 mg/kg was capable of reducing the weight loss in treated mice. The treatment with both doses also reduced the cellular migration and reduced IL-6 levels in peritoneal exudate and serum in doses of 20 and 10 mg/kg, respectively. They also presented a protective effect in the liver, with a reduction in hepatic transaminase levels in both doses of treatment and attenuated histological damage in the liver at a dose of 10 mg/kg. Fucan from *S. schröederi* presented a promising pharmacological activity upon the murine model of ZIGI, with potential anti-inflammatory and hepatic protective effects, and should be the target of profound and elucidative studies.

## 1. Introduction

Fucans are sulfated polysaccharides found in brown algae (*Phaeophyceae*) and echinoderms (sea urchins and sea cucumbers). The family of algae fucans can be divided into three big groups of polysaccharides: fucoidans (mostly consisting of fucose), xylofucoglucuronans, and glucuronogalactofucans (or glucurofucogalactanans) [1]. The structure of these sulfated polysaccharides changes according to the algae species from which they are extracted, and this structure is intrinsically related to their biological activity [2].

Since their first description, fucans from marine organisms have been the target of many studies, showing a broad spectrum of biological properties: antitumoral, antiproliferative [3,4], anticoagulant, antithrombotic [5,6], hypoglycemic [7], antiviral [8], antiparasitary [9], antioxidant [10], immunoregulatory [11], and anti-inflammatory [12]. Marine algae are a source of many compounds with biological activity, including polysaccharides with anti-inflammatory activity [12]. Fucans with anti-inflammatory activity were found in brown seaweeds [13,14], turning these organisms into an interesting source of biological compounds for study. Brown algae *Spatoglossum* spp. are well described as a source of compounds with many biological activities [15,16,17]; among them, *Spatoglossum schröederi*, a commonly brown seaweed found on the Brazilian coast, show antigenotoxic [18], antitumoral [19], anti-adhesive [20], antithrombotic [21], antigenotoxic [18], antinociceptive [22], anti-angiogenic [23], antiparasitic [24], and nutraceutical [25] effects. Despite the variety of studies that characterize this seaweed as a source of many compounds with potential pharmacological activities, there is a lack of knowledge and prospective studies about its anti-inflammatory potential.

Systemic inflammatory response, like sepsis, can lead to conditions that could seriously impact the body function and, in some cases, the survival of affected individuals. Sepsis is defined as a “life-threatening organ dysfunction caused by a dysregulated host response to infection,” according to the more recent guidelines [26]. Sepsis pathophysiology involves many immunological mechanisms, with intense production of pro-inflammatory factors, mainly TNF-α, IL-1β, IL-6, IL-12, and IL-18, in a marked event called “cytokine storm” [27], leading to intense systemic inflammatory activation, causing tissue damage and organ dysfunction [28]. Sepsis and septic shock diagnosis is based on the assessment of clinical scores, laboratory biomarkers of inflammatory response, circulatory and metabolic dysregulation, and progressive organ dysfunction [29,30], which is associated with a higher risk of mortality in septic patients [26].

Due to the complex immunopathogenesis and clinical manifestation, sepsis is a condition of difficult management and treatment, requiring hemodynamic stabilization and vasopressor therapy to avoid shock and antibiotic therapy to eliminate infectious focuses as major strategies of disease control [31]. Immunosuppressor therapy with corticosteroids has been established as a management strategy, but studies are very controversial about the efficacy of these drugs in sepsis control [32]. In the same way, many clinical trials have failed to establish a successful anti-inflammatory pharmacological therapy as a fully effective strategy in sepsis control [33,34]. Therefore, sepsis remains one of the major healthcare challenges worldwide, with a high mortality rate mainly in undeveloped regions [35].

In addition to clinical trials to establish effective anti-inflammatory therapeutic strategies, preliminary studies are always necessary for a better understanding of the immunopathogenesis of sepsis to evaluate and propose new potential intervention protocols and treatment strategies. Non-septic shock induced by zymosan was proposed decades ago as a study model of generalized inflammation (denominated as zymosan-induced generalized inflammation—ZIGI), which can trigger multiple-organ dysfunction in mice [36] in a similar way observed in humans stricken with sepsis. Zymosan is a non-degradable polysaccharidic compound from the *Saccharomyces cerevisiae* cell wall capable of inducing macrophagic activation and synthesis of many pro-inflammatory mediators, such as cytokines, bioactive lipids, and oxygen radicals, leading to long-term inflammation status [37]. The ZIGI model is marked by an intense inflammatory status with clinical, biochemical, and histological changes, inducing systemic toxicity and weight loss [38]; intense leukocyte migration, with augmented polymorphonuclear infiltrate [39]; increased levels of TNF-α, IL-1β, and IL-6 and mediators of oxidative stress, associated with the NF-κB role of immune modulation [40,41]; and important histopathological changes in affected tissues and biochemical-associated manifestations, like liver damage and augmented hepatic transaminases levels [42,43].

Considering the lack of anti-inflammatory therapeutical strategies for sepsis and the gravity of this condition, the importance of conducting studies with the purpose of elucidating new pharmacological intervention possibilities has emerged. The present work demonstrates the biological activity of a fucan extracted from the brown seaweed *Spatoglossum schröederi* as a treatment for systemic inflammation in a ZIGI murine model. This is a pioneer study to evaluate the potential anti-inflammatory effect of a fucan extracted from this brown seaweed, applying an experimental model that reproduces similar immunopathogenesis observed in organ dysfunction triggered by a systemic inflammatory response.

## 2. Results

### 2.1. Fucan from S. schröederi Can Inhibit Leukocyte Migration to Peritoneal Cavity in Zymosan-Induced Peritonits Murine Model

**Figure 1:**
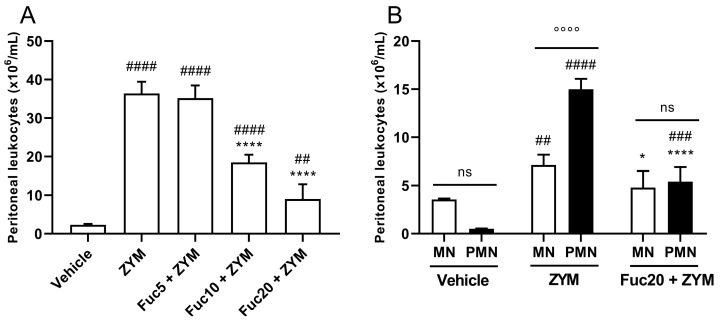
Effect of treatment with fucan from *S. schröederi* in total (**A**) and differential (**B**) leukocyte migration to the peritoneal cavity in zymosan-induced peritonitis murine model. BALB/c mice were treated intravenously (i.v.) with three different doses of fucan, and, 1 h later, they were inoculated intraperitoneally (i.p.) with zymosan (40 mg/kg). The negative control group (vehicle) received NaCl 0.9% as a challenge and treatment. The positive control group (ZYM) received zymosan as a challenge and NaCl 0.9% as treatment. After 24 h, peritoneal exudate was collected, and total and differential cellularity were determined. The data are representative of three independent experiments (*N* = 5 animals per group) and are presented as mean with standard deviation. In (**A**), **** *p* < 0.0001, when compared with the positive control group. #### *p* < 0.0001 and ## *p* = 0.0086 when compared with the negative control group. In (**B**), **** *p* < 0.0001 and * *p* = 0.0194, when compared with PMN and MN from the positive control group, respectively; #### *p* < 0.0001 and ### *p* = 0.0007, when compared with PMN from the negative control group; ## *p* = 0.0069, when compared with MN from the negative control group; °°°° *p* < 0.0001. Fuc20: fucan 20 mg/kg. Fuc10: fucan 10 mg/kg. Fuc5: fucan 5 mg/kg. ZYM: zymosan; MN: mononuclear cells; PMN: polymorphonuclear cells. ns, no significant difference between selected group.

First, the biological activity of the compound was evaluated in zymosan-induced peritonitis, a simple model of acute inflammation, to determine if a fucan extracted from *S. schröederi* has any anti-inflammatory potential. In this model, zymosan can induce intense leukocyte migration to the peritoneal cavity 24 h after its inoculation. Treatment showed dose-dependent inhibition of leukocyte migration, with a marked effect at doses of 20 and 10 mg/kg, while the fucan dose of 5 mg/kg did not show any effect (Figure 1A). The main cell type present in the inflammatory infiltrate at the time studied after zymosan inoculation was polymorphonuclear cells (PMNs), followed by mononuclear cells (MNs). Treatment with a dose of 20 mg/kg of fucan from *S. schröederi* significantly reduced the presence of these cells in the inflammatory infiltrate, reducing the proportion of MNs to a status similar to that observed in healthy animals (Figure 1B).

### 2.2. Fucan from S. schröederi Can Attenuate Systemic Toxicity Signs in ZIGI Murine Model

**Figure 2:**
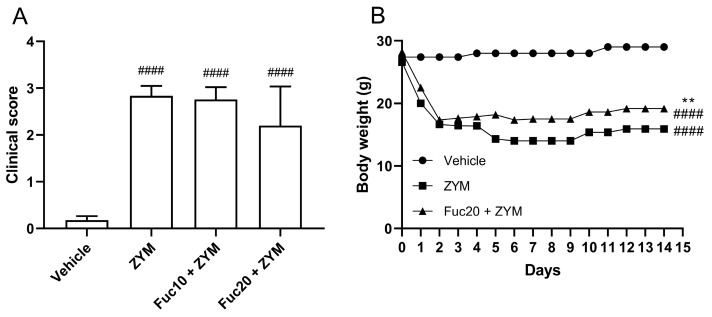
Effect of treatment with fucan extracted from *S. schröederi* in systemic toxicity in the ZIGI murine model. BALB/c mice were treated with fucan (i.v.) 1 h before and 6 h after zymosan challenge (500 mg/kg, i.p.). The negative control group (vehicle) received NaCl 0.9% as a challenge and treatment. The positive control group (ZYM) received zymosan as a challenge and NaCl 0.9% as treatment. (**A**) Clinical signs of generalized inflammation (bristly hair, prostration, and diarrhea) were evaluated 6 h after zymosan administration. Each clinical sign was attributed a score of 1 (presence) or 0 (absence). (**B**) Body weight was recorded daily for 15 days after zymosan administration. The data are representative of three independent experiments (*N* = 5 animals per group in A; *N* = 10 animals per group in B) and are presented as mean with standard deviation. In (**A**,**B**): #### *p* < 0.0001, when compared with the negative control group. In (**B**): ** *p* = 0.0084, when compared with the positive control group. Fuc20: fucan 20 mg/kg. Fuc10: fucan 10 mg/kg. ZYM: zymosan.

Since the fucan from *S. schröederi* showed a potent inhibitory effect on leukocyte migration in the peritonitis model, its activity was also evaluated in the ZIGI model. After observing the inefficacy of a fucan dose of 5 mg/kg to impair leukocyte migration in the initial experiment, it was decided to test only the other two doses in subsequent steps. The impact of the treatment on attenuating the clinical signs, such as bristly hair, prostration, and diarrhea, as well as body weight loss, was evaluated, which is a criterion for evaluating systemic toxicity in animals subjected to generalized inflammation. Zymosan was able to induce important systemic toxicity in the generalized inflammation model, with a high clinical score (Figure 2A) and accented body weight loss (Figure 2B) in affected animals. Treatment with fucan was not able to reduce clinical signs of systemic toxicity in this model. Animals treated with fucan were also followed up for 15 days after zymosan inoculation. We tested the treatment with 20 mg/kg of fucan, as this dose showed better results in the peritonitis model. In the first 18 h, no difference in the body weight loss was observed between experimental groups. However, 24 h after zymosan administration, a discreet but significant reduction in body weight loss was registered in the treatment group, which remained throughout the analyzed period.

### 2.3. Fucan from S. schröederi Can Inhibit Peritoneal Leukocyte Migration in ZIGI Murine Model

**Figure 3:**
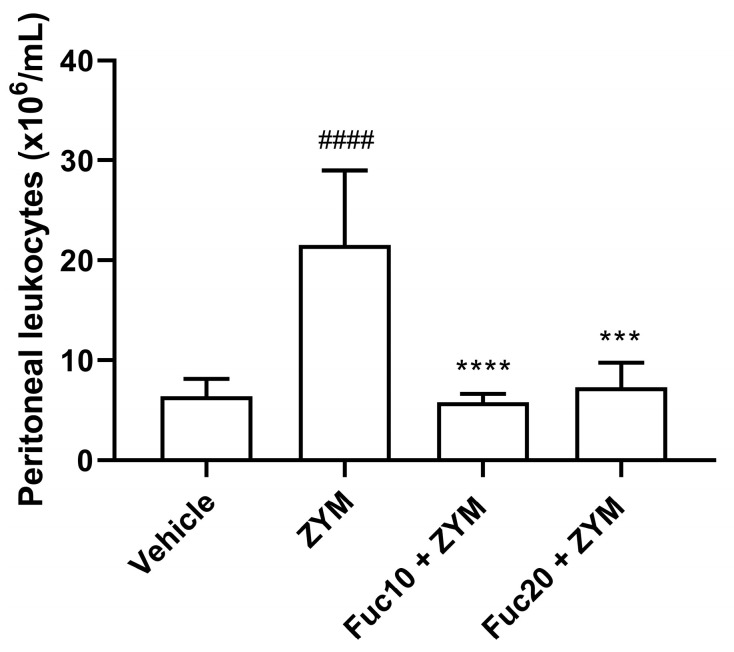
Effect of treatment with fucan extracted from *S. schröederi* in leukocyte migration to the peritoneal cavity in the ZIGI model. BALB/c mice were treated with fucan (i.v.) 1 h before and 6 h after zymosan challenge (500 mg/kg, i.p.). The negative control group (vehicle) received NaCl 0.9% as a challenge and treatment. The positive control group (ZYM) received zymosan as a challenge and NaCl 0.9% as treatment. Eighteen hours after zymosan administration, peritoneal exudate was collected, and total cellularity was determined in the Neubauer chamber. The data are representative of three independent experiments (*N* = 5 animals per group) and are presented as mean with standard deviation. **** *p* < 0.0001 and *** *p* = 0.0001 when compared with the positive control group. #### *p* < 0.0001, when compared with the negative control group. Fuc20: fucan 20 mg/kg. Fuc10: fucan 10 mg/kg. ZYM: zymosan.

Our next step was to evaluate the effect of doses of 20 and 10 mk/kg of fucan on cell migration to the peritoneum of animals submitted to the generalized inflammation model induced by zymosan. As expected, the animals submitted to the ZIGI model showed intense leukocyte infiltration in the peritoneum. On the other hand, treatment with fucan, at the two doses tested, showed a potent anti-inflammatory effect, reducing cell count to similar levels observed in healthy animals (Figure 3).

### 2.4. Fucan from S. schröederi Can Reduce IL-6 Levels in ZIGI Murine Model

**Figure 4:**
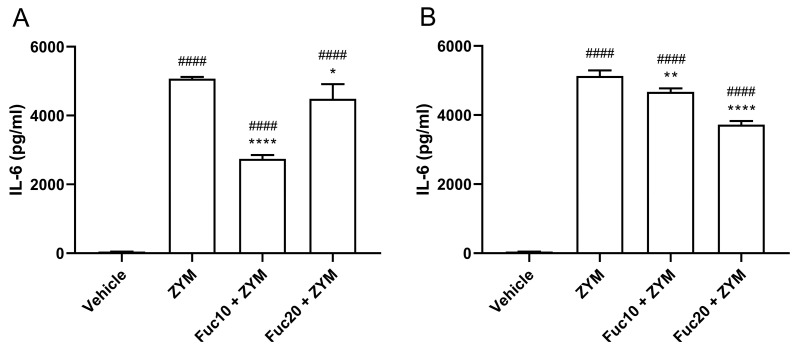
Effect of treatment with fucan extracted from *S. schröederi* in IL-6 levels in serum (**A**) and peritoneal exudate (**B**) in the ZIGI model. BALB/c mice were treated with fucan (i.v.) 1 h before and 6 h after the zymosan challenge (500 mg/kg, i.p.). The negative control group (vehicle) received NaCl 0.9% as a challenge and treatment. The positive control group (ZYM) received zymosan as a challenge and NaCl 0.9% as treatment. Eighteen hours after zymosan administration, sera, and peritoneal exudate were collected, and IL-6 levels were determined by ELISA. The data are representative of three independent experiments (*N* = 5 animals per group) and are presented as mean with standard deviation. **** *p* < 0.0001, ** *p* = 0.002, and * *p* = 0.0293, when compared with the positive control group. #### *p* < 0.0001, when compared with the negative control group. Fuc20: fucan 20 mg/kg. Fuc10: fucan 10 mg/kg. ZYM: zymosan.

Next, the efficacy of treatment with fucan to modulate IL-6 levels in serum and peritoneum exudate in animals submitted to ZIGI was determined. Animals subjected to the ZIGI model showed high levels of IL-6 both in blood serum (Figure 4A) and in peritoneal exudate (Figure 4B) as a sign of important pro-inflammatory response. In sera, treatment with fucan with the two tested doses reduced cytokine levels, with a dose of 10 mg/kg showing a better effect than the higher dose of treatment (20 mg/kg). In the same way, IL-6 levels in peritoneal exudate were also reduced with doses of 10 and 20 mg/kg. However, in this case, the higher dose presented better results in modulating cytokine levels. In both experiments, however, treatment was able to attenuate but did not reduce IL-6 to normal levels when compared with the negative control group.

### 2.5. Fucan of S. schröederi Show Protective Hepatic Effect, Attenuating Liver Damage in ZIGI Murine Model

**Figure 5:**
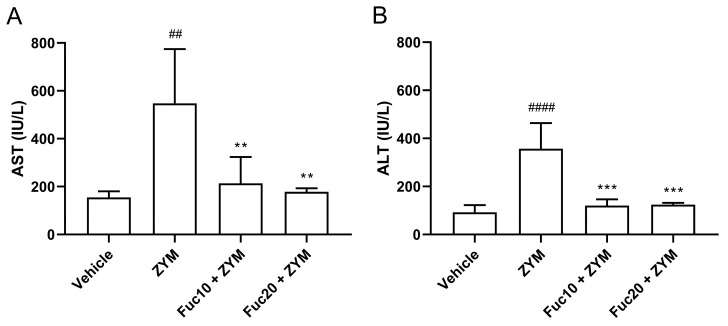
Effect of treatment with fucan extracted from *S. schröederi* in AST (**A**) and ALT (**B**) in the ZIGI model. BALB/c mice were treated with fucan (i.v.) 1 h before and 6 h after zymosan challenge (500 mg/kg, i.p.). The negative control group (vehicle) received NaCl 0.9% as a challenge and treatment. The positive control group (ZYM) received zymosan as a challenge and NaCl 0.9% as treatment. Eighteen hours after zymosan administration, sera were collected, and transaminase levels were determined by biochemical photometric assay. The data are representative of three independent experiments (*N* = 5 animals per group) and are presented as mean with standard deviation. *** *p* = 0.0002, ** *p* = 0.0077 (Fuc10 + ZYM), and ** *p* = 0.0038 (Fuc20 + ZYM) when compared with the positive control group, ## *p* = 0.0024 and #### *p* < 0.0001, when compared with negative control group. Fuc20: fucan 20 mg/kg. Fuc10: fucan 10 mg/kg. ZYM: zymosan.

Finally, the effect of treatment with fucan on hepatic damage observed in the ZIGI model was evaluated. Initially, aspartate (AST) and alanine (ALT) aminotransferase levels in sera were assessed. The ZIGI model was proven to induce high levels of AST (Figure 5A) and ALT (Figure 5B) in animals as important biochemical signs of liver dysfunction in zymosan-induced generalized inflammation. Treatment with fucan in two tested doses was able to reduce hepatic transferase levels to normal status without a difference between treated and healthy groups. Interestingly, no difference in the protective effect was observed between both doses in this experiment.

**Figure 6:**
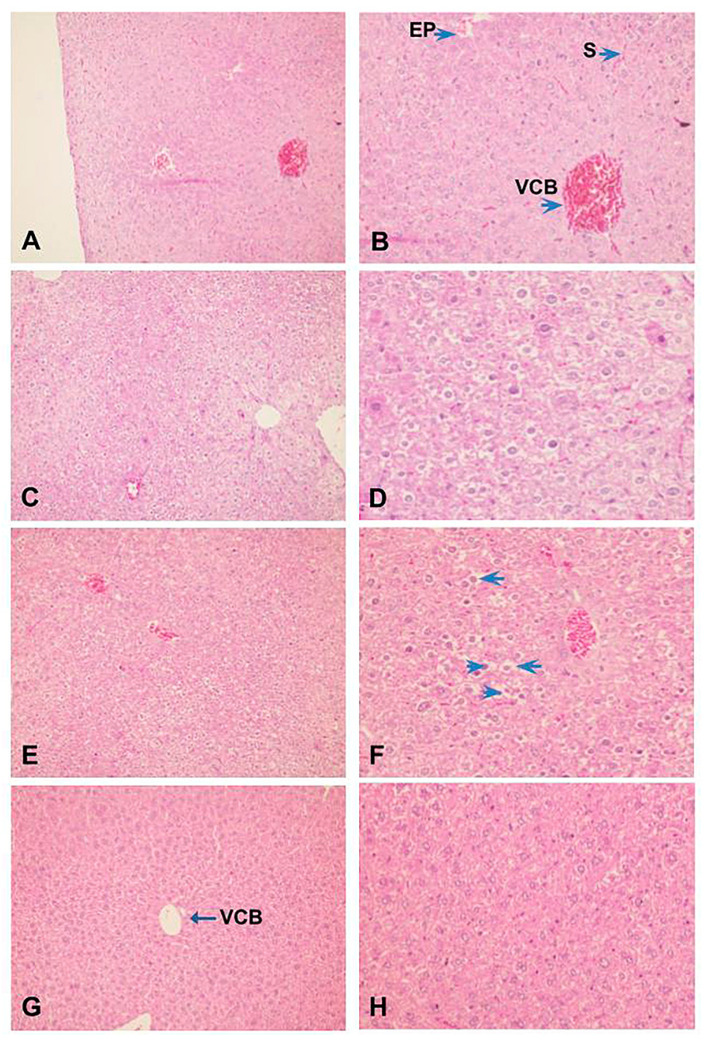
Effect of treatment with fucan extracted from *S. schröederi* in histopathological hepatic changes in the ZIGI model. BALB/c mice were treated with fucan (i.v.) 1 h before and 6 h after zymosan challenge (500 mg/kg, i.p.). The negative control group received NaCl 0.9% as a challenge and treatment. The positive control group received zymosan as a challenge and NaCl 0.9% as a treatment. Eighteen hours after zymosan administration, animals were euthanized, and the liver was collected for histopathological analysis. Samples were fixed and colored by H/E. (**A**) Negative control group (100×). (**B**) Detail from hepatic parenchyma of the negative control group (200×). (**C**) Positive control group (100×). (**D**) Detail from the liver peripheric area of the positive control group showing hepatocytes with irreversible alterations: apoptosis, ballooning degeneration, and hemorrhagic leakage areas (200×). (**E**) Treatment with fucan from *S. schröederi*, a dose of 20 mg/kg (100×). (**F**) Detail of hepatocytes in E, showing the presence of nuclear pyknosis (arrowhead) and cytoplasmatic vacuolization (arrow) (200×). (**G**) Treatment with fucan from *S. schröederi*, a dose of 10 mg/kg (100×). (**H**) Detail of hepatocytes in G, showing discrete alterations compatible with regeneration areas. VCB: central lobular vein. EP: portal space. S: sinusoid.

Also, histopathological analysis of the liver showed that ZIGI can induce severe tissue damage, parenchyma disorganization, extensive necrose areas, hemorrhagic leakage, presence of hepatocyte degeneration, nuclear pyknosis, and chromatin fragmentation (Figure 6C,D). Treatment with fucan from *S. schröederi* reduced liver damage, presenting discrete hepatocyte alterations without parenchyma disorganization. The protective effect is more expressive in the group treated with 10 mg/kg of fucan (Figure 6G,H), presenting the absence of hemorrhagic focuses, hepatocyte cord integrity preserved, and discrete alterations compatible with tissue regeneration. In the group treated with 20 mg/kg of fucan, it was possible to observe the presence of congested vessels and sinusoids, parenchyma disorganization, presence of nuclear pyknosis, and cytoplasmatic vacuolization in hepatocytes (Figure 6E,F).

## 3. Discussion

Fucans are sulfated polysaccharides commonly found in brown seaweed, with a range of biological effects and potential use in medicine [44,45]. The anti-inflammatory activity of fucans has been studied, with many immunomodulatory mechanisms described [46], but, as previously mentioned, there is a lack of knowledge about the anti-inflammatory effect of fucan extracted from *Spatoglossum* spp. The evidence presented showed a broad anti-inflammatory effect of fucan extracted from the brown seaweed *Spatoglossum schröederi* in zymosan-induced generalized inflammation, a model of severe inflammatory injury capable of triggering organ dysfunction and highlighted some characteristics of the biological activity of this compound.

Marine polysaccharides, including fucans, have been recognized for their diverse bioactivities, which encompass anti-inflammatory effects. For example, sulfated fucans from brown algae have demonstrated anti-inflammatory potential by modulating various cellular and molecular components of the immune system [47]. These polysaccharides can interact with immune cells, such as macrophages and neutrophils, inhibiting chemotaxis and adhesion to endothelial cells [48]. In fact, a fucan obtained from brown seaweed reduced swelling and paw volume in the paw edema murine model [49,50]. Fucan from brown seaweed was proven to impair cellular migration through binding cell adhesion molecules [51]. In fact, fucan from *S. schröederi* was proven to inhibit the migration of CHO-K1 cells [23]. Also, fucan extracted from *Saccharina japonica* was proved to be capable of reducing mRNA CD11b levels and CD11b expression [52], impacting leukocyte migration under pro-inflammatory conditions. According to this, a similar effect was observed in this study when fucan from *S. schröederi* was able to inhibit cellular migration not only in acute inflammation conditions, during peritonitis experiments, but also in severe inflammatory response, as in the ZIGI model. The data suggest, for the first time, an important effect of these compounds in inhibiting cellular migration during generalized inflammation.

Additionally, the capacity of fucans to reduce polymorphonuclear (PMN) migration is noteworthy. PMNs are key players in the early stages of inflammation, but excessive PMN recruitment can exacerbate tissue damage [53]. Indeed, sulfated fucan extracted from Padina gymnospora was proved to be capable of reducing the peritoneal PMN migration in the acute peritonitis model [54]. Fucoidan from *Sargassum hemyphillum* was also able to drastically reduce PMN migration to the lungs in pneumonitis and the fibrosis model in mice, an effect associated with diminished pro-inflammatory cytokine production, attenuating tissue damage [55]. The ability of fucan to markedly reduce PMN migration, as shown in our study, aligns with its potential as an anti-inflammatory agent that may help prevent excessive inflammation-induced tissue damage.

Interestingly, inhibition of leukocyte migration to the injury site (and attenuating of other parameters, as discussed below) by fucan from *S. schröederi* was observed in this study in a dose-dependent manner. Dose-dependent effects of fucans have been reported in different experimental settings upon various parameters, such as reduction in exudate leakage, diminished pro-inflammatory cytokine, ROS and NO production, modulation of enzyme activity associated with inflammation, and inhibition of signaling pathway responsible for pro-inflammatory gene expression [49,56,57]. These findings allow us to modulate experiments by choosing lower but effective doses and avoiding the possibility of toxicity. It is also important to consider that the efficacy of fucans can be influenced by their molecular weight, sulfation pattern, and degree of branching [48,58]. This complexity in their structure may contribute to the observed dose-dependent response and must be considered in prospecting future studies for a better evaluation of the anti-inflammatory activity of this compound.

Besides leukocyte migration, treatment with fucan from *S. schröederi* was also capable of attenuating systemic toxicity in the ZIGI model. A similar effect was observed in the DSS-induced colitis murine model when the treatment with fucan from the brown seaweed *Macrocystis pyrifera* ameliorated global clinical signs and disease activity index, including body weight loss in an anti-inflammatory effect associated with the diminished production of pro-inflammatory cytokines [59]. Our data show that treatment with fucan from *S. schröederi* in the ZIGI model was also capable of reducing body weight loss, an important parameter of systemic toxicity evaluation, and this effect was also accompanied by diminished IL-6 levels in peritoneal exudate and sera. Importantly, fucan from *S. schröederi* was proved here to positively modulate the clinical status in ZIGI; the model chosen here underscores the severity of the inflammatory response and serves as a challenging testbed for potential anti-inflammatory interventions.

One noteworthy finding is that fucan treatment, administered at a dosage of 20 mg/kg, did not effectively reduce the clinical signs of systemic toxicity within the first 18 h of observation. This result is consistent with the study by Kuznetsova et al. [60], which showed that pretreatment with fucoidan extracted from brown algae, *Fucus evanescens*, used in a murine model of endotoxemia caused by LPS, partially restored the hypercoagulopathy, characteristic of this inflammatory condition. However, the delayed but significant reduction in body weight loss observed 24 h after zymosan administration in the fucan-treated group suggests a potential time-dependent effect of fucan. Again, our data corroborate the previously cited study, which shows that pretreatment with fucoidan prolonged the survival of the animals by days.

Treatment with fucan from *S. schröederi* was also capable of reducing IL-6 levels in peritoneal leakage and serum in the ZIGI model, demonstrating the ability of the compound to modulate the cytokine levels locally at the site of injury and systemically. IL-6 is an important pro-inflammatory cytokine involved in many immune response mechanisms, such as leukocyte proliferation and recruitment and acute phase protein and antibody production, displaying a central role in the pathogenesis of severe diseases [61]. During systemic inflammation, IL-6 can enhance TNF-α production via NF-κB signaling activation [62], which can create conditions for cytokine storm onset and associated organ damage. Notably, increased IL-6 systemic levels were associated with severe inflammatory response in patients with sepsis [63] and have an important predictive role as a multiple-organ dysfunction biomarker [64]. Remarkably, the impact of treatment with fucan from *S. schröederi* on IL-6 levels is a considerable find of this study due to the relationship of high levels of this pro-inflammatory factor with the pathogenesis of organ failure.

This inhibitory effect of fucans upon pro-inflammatory cytokines was also observed in other studies. Fucans obtained from brown seaweed have been shown to reduce pro-inflammatory cytokine levels, such as IL-1β, IL-6, IL-8, and TNF-α, with no sign of toxicity or cellular viability loss [65,66]. A similar effect was registered in in vitro and in vivo experiments with fucan extracted from *Sargassum fusiforme*, reducing not only pro-inflammatory cytokine levels but also NO and PGE_2_ levels, and this was associated with NF-κB signaling modulation [67]. A fucan obtained from brown seaweed has been shown to have anti-inflammatory activity associated with the regulation of central signaling pathways, like MAPK and NF-κB, reducing phosphorylation of p38, ERK, JNK, p65, and IKKα/IKKβ [56,68], and NF-κB nuclear translocation [49] in in vitro and in vivo experiments.

NF-κB and MAPK pathways are pivotal in pro-inflammatory and immunomodulatory signaling and are responsible for gene expression of many mechanisms and factors of immune response, such as chemokines, cytokines, signaling proteins and receptors, cellular adhesion molecules, and oxidative stress mediators [69,70,71]. In vitro and in vivo studies with fucans from brown seaweed showed the capacity of these compounds to inhibit NF-κB and MAPK pathways, downregulating pro-inflammatory factors such as iNOS, COX-2, and MyD88, and attenuating the production of mediators such as TNF-α, IL-6, Il-1β, NO, and PGE_2_ [12,72,73,74,75]. Considering the extensive evidence and the characteristics of the anti-inflammatory effect of fucan from *S. schröederi* elicited by our experiments, it is hypothesized that this compound can show similar activity. Unfortunately, it was not possible to evaluate the molecular mechanisms of the anti-inflammatory activity of fucan from *S. schröederi* in the present work and confirm the existence of this immunomodulating mechanism by the compound.

As discussed before, systemic inflammatory response has a major role in the onset of tissue damage due to the capacity of recruitment and activation of leukocytes (mainly PMN) and the production of pro-inflammatory mediators responsible for immune-mediated aggression and organ dysfunction. Treatment with these compounds was proven to prevent damage in the lung and intestinal colon by inhibiting the inflammatory response during severe diseases, reducing leukocyte infiltration, pro-inflammatory cytokine production, tissue fibrosis, and relative clinical manifestation [59,76]. Fucans and sulfated polysaccharides from marine algae have also demonstrated hepatoprotective effects through anti-inflammatory mechanisms in various liver injury models. They reduce hepatic transaminase levels, lower pro-inflammatory mediator amounts, and preserve tissue structure and function [77,78,79]. Importantly, liver function impairment represents a critical event during multiple-organ dysfunction pathogenesis, with a bad outcome for the patient due to the importance of hepatic functions to the entire organism [80,81] and, in this context, the hepatoprotective effect presented by fucan from *S. schröederi*, elicited by biochemical and histopathological analysis, rises as one of the most important findings of our work, and this effect was attributed to the anti-inflammatory activity shown by the compound in our experiments.

Our study has some limitations, such as the lack of evidence about the molecular mechanisms responsible for the anti-inflammatory activity of fucan from *S. schöederi*. More studies are necessary to elucidate these mechanisms, allowing us to discuss with more propriety the range of its pharmacological potential and bioprospection perspectives. It is important to highlight the importance of the data presented in this study due to the lack of knowledge about the biological activity of metabolites from *S. schröederi*, mainly the anti-inflammatory of fucans and sulfated polysaccharides from this species, to aim for more effective therapeutics for immune-mediated severe diseases such as generalized inflammation and multiple-organ dysfunction.

## 4. Materials and Methods

### 4.1. Animals

Six- to eight-week-old BALB/c male mice with 20–25 g of body weight were used in this study. Mice were obtained from the Biosciences Center Bioterium of the Federal University of Rio Grande do Norte. Mice were maintained in a 12 h/12 h light–dark cycle, with free access to food and water. All procedures in this study were authorized by the Experimental Animals Ethical Committee of the Bioscience Center of the Federal University of Rio Grande do Norte (Protocol 008/2010).

### 4.2. Fucan Obtaining

*Spatoglossum schöederi* seaweed was collected at Pirambuzios Beach (5°59′20.6″ S 35°06′50.1″ W), Nísia Floresta-RN, Brazil. It was preserved in the Natural Polymers Biotechnology Laboratory—BIOPOL, in the Department of Biochemistry of the Federal University of Rio Grande do Norte, supervised by Prof. Dr. Hugo Alexandre de Oliveira Rocha. The collection was authorized by the Brazilian National Management System for Genetic Heritage and Associated Traditional Knowledge (SISGEN number A0D4240).

The seaweeds were dried at 50 °C with ventilation, blended, and treated with ethanol to remove lipids and pigments. Several changes of ethanol were performed, and by the fifth time, even after 24 h of maceration, the ethanol was no longer pigmented. Subsequently, the material was centrifuged at 8000× *g*, 4 °C for 30 min, and the resulting seaweed powder was dried at room temperature while being shielded from light. This material was then stored in sealed containers and kept away from light until the fucan extraction process.

The fucan was extracted from marine brown algae *S. schröederi* following the methodology outlined in the study by Rodrigues-Souza et al. [18]. Approximately 100 g of powdered algae was suspended in five volumes (500 mL) of 0.25 M NaCl, and the pH was adjusted to 8.0 using NaOH. Subsequently, 1.5 g of Prolav 750 (Prozyn Biosolutions, São Paulo, Brazil), which is a mixture of alkaline proteases, was added to facilitate proteolytic digestion. After 18 h incubation at 60 °C, the mixture was filtered through cheesecloth. The resulting solution was termed the crude extract and underwent acetone fractionation.

The crude extract was subjected to acetone precipitation as follows: ice-cold acetone (0.5 mL) was gently added to the solution and maintained at 4 °C for 24 h. The resulting precipitate was collected by centrifugation (10,000× *g*, 20 min), dried under vacuum, reconstituted in distilled water, and subjected to analysis. This process was repeated by adding 0.6, 0.7, 0.9, 1.1, 1.3, and 2.0 volumes of acetone to the supernatant. These fractions were named based on the volume of acetone used: F0.5v, F0.6v, F0.7v, F0.9v, F1.1v, F1.3v, and F2.0v.

The F0.6v fraction, which was confirmed to contain fucan based on the findings of Rodrigues-Souza et al. [18], was dissolved in distilled water and subjected to ion exchange chromatography. The elution process involved a gradient of increasing NaCl concentrations (0.25/0.5/0.7/1.0/1.5/2.0 M). The fraction eluted with 1.0 M NaCl, known to contain the fucan, was precipitated by adding methanol (100%) and kept at 4 °C. After 24 h, the precipitate was separated by centrifugation, dialyzed, and stored in light-protected conditions for future analyses. Fucan identification was performed through 1HNMR analysis, and the corresponding spectra can be found in their previously published paper.

### 4.3. Zymosan-Induced Peritonitis

BALB/c mice were randomly distributed in study groups (*N* = 5 animals per group) and inoculated by intravenous route with sterile saline solution (0.9%) or different doses of fucan (Fuc5: fucan 5 mg/kg; Fuc10: fucan 10 mg/kg; Fuc20: fucan 20 mg/kg) diluted in sterile saline solution (0.9%) as treatment. After 1 h, the negative control group (vehicle) received by intraperitoneal route 500 µL of sterile saline solution (0.9%), while the positive control (ZYM) and treated (Fuc5, Fuc10, and Fuc20) groups received 500 µL of zymosan solution (40 mg/kg) by the intraperitoneal way as a stimulus. After 24 h of stimulus inoculation, animals were euthanized, and peritoneal lavage was collected by injection of 5 mL of cold sterile saline solution (0.9%). The recovered material was centrifuged (250× *g*, 10 min, 4 °C), and the supernatant was collected by IL-6 determination assays. The cellular button was resuspended in cold saline solution (0.9%), and the cellular count was determined by optical microscopy with a Neubauer chamber.

### 4.4. Zymosan-Induced Generalized Inflammation Model (ZIGI)

ZIGI was performed using the methodology described by Paola et al. [38], with modifications. BALB/c mice were randomly distributed in study groups (*N* = 5 animals per group). Generalized inflammation was induced by intraperitoneal administration of zymosan (500 mg/kg) diluted in sterile saline solution (0.9%) as a stimulus. The negative control group (vehicle) received sterile saline solution only as a stimulus. One hour before and six hours after the stimulus, animals were treated intravenously with different doses of fucan (Fuc10: fucan 10 mg/kg; Fuc20: fucan 20 mg/kg) diluted in sterile saline solution (0.9%). Negative (vehicle) and positive (ZYM) control groups received sterile saline solution (0.9%) only as treatment. After 18 h of stimulus inoculation, mice were anesthetized, blood was collected by retro-orbital punction and centrifuged (1400× *g*, 10 min, 4° C), and sera were preserved for IL-6 and hepatic transaminases determination. Next, animals were euthanized, peritoneal lavage was collected as previously described, and the liver was extracted and preserved in PBS-formalin solution (10%) for posterior histopathological analysis.

### 4.5. IL-6 and Hepatic Transaminase (ALT/AST) Determination

IL-6 concentration in sera and peritoneal lavage supernatant was determined by enzyme-linked immunosorbent assay (ELISA), according to the manufacturer’s recommendations (eBioscience., Inc., San Diego, CA, USA). ALT and AST levels in sera were determined according to the manufacturer’s recommendations (Labtest Diagnóstica S.A., Lagoa Santa, Brazil).

### 4.6. ZIGI Systemic Toxicity Evaluation

The severity of systemic injury occasioned by ZIGI was also analyzed according to Paola et al. (2006) methodology. Generalized inflammation clinical signs (bristly hair, prostration, and diarrhea) were subjectively observed in animals 6 h after stimulus inoculation. A number score was attributed to each clinical sign observed, with 0 for absence and 1 for presence of the parameter analyzed, and the total score ranges from 0 to 3 for each animal observed. In another experimental series, animals submitted to the ZIGI model were treated with fucan at a dose of 20 mg/kg and were monitored and weighed daily for 15 days to evaluate the loss of body weight induced by generalized inflammation.

### 4.7. Histopathological Analysis

The collected liver quantitative samples were fixed in PBS-formalin solution (10%) and then arranged in paraffin blocks. Paraffin blocks were cut to obtain 5 µm thick slices of tissue for hematoxylin–eosin coloration. Morphological analysis was evaluated in a blind study of cases performed by one single evaluator (E. J. D. S.), who registered and described qualitatively the histological status of tissue parenchyma and stroma.

### 4.8. Data Analysis

The data were tabled and expressed as mean with standard deviation in every graphical representation. Statistical analysis was performed by application of ANOVA with Dunnet and Sidak post hoc tests to determine the difference between experimental groups. Statistical significance was considered when *p* < 0.05. All graphical representation and statistical analysis were performed using GraphPad Prism 8.0.1 software (GraphPad Software, Inc., La Jolla, CA, USA).

## 5. Conclusions

This is the first study to show the anti-inflammatory effects of fucan extracted from the brown seaweed *Spatoglossum schröederi* in murine models of acute peritonitis and generalized inflammation induced by zymosan. The data show the capacity of fucan to inhibit zymosan-induced leukocyte migration to the peritoneum, with a remarkable ability to impair polymorphonuclear migration, specifically. Fucan from *S. schröederi* was also able to ameliorate the clinical manifestation of the ZIGI model, reducing body weight loss, an important parameter of systemic toxicity evaluation. Additionally, treatment with fucan was capable of reducing IL-6 at local and systemic levels, in addition to having a hepatoprotective effect, reducing liver damage and hepatic transaminase levels. Considering these findings, more studies are necessary to investigate the mechanisms responsible for the biological activity of this compound and characterize this fucan as a potential resource in the treatment of severe inflammatory diseases.

## Data Availability

The original data presented in the study are included in the article. Further inquiries can be directed to the corresponding author.
